# Malignancy rates and initial management of Thy3 thyroid nodules in a district general hospital: The ‘Reading’ experience

**DOI:** 10.1002/edm2.243

**Published:** 2021-02-24

**Authors:** Dilip Nair, Shivanthi Kandiah, Thomas Rourke, Rogan Corbridge, Sidhartha Nagala

**Affiliations:** ^1^ Department of ENT Royal Berkshire NHS Foundation Trust Hospital Reading Berkshire UK

**Keywords:** fine‐needle aspiration cytology, histology, rate of malignancy, Thy3 thyroid nodules

## Abstract

**Background:**

Ultrasound‐guided fine‐needle aspiration cytology is the gold standard for investigating thyroid nodules. Stratifying the Thy3 thyroid nodule risk of malignancy is essential for clinical decision‐making. According to the Royal College of Pathologists Guidance (2016), the rate of malignancy for Thy3a is 5–15% and for Thy3f 15–30%. Our aim was to investigate the malignancy rate and the initial management of Thy3 nodules in our institution.

**Methods:**

A retrospective review was undertaken of 115 patients with Thy3 cytology results from thyroid fine‐needle aspirations performed between January 2015 and June 2020 at a single centre. A total of 90 out of 115 patients underwent surgery.

**Results:**

Of the 90 patients, we had a 40% malignant rate (36/90). Specifically, 14 of 34 (41.1%) Thy3a and 22 of 56 (39.2%) Thy3f nodules were malignant. Of the malignant lesions, 52.7% (19/36) were follicular thyroid carcinoma. 58.8% (10/17) of male patients and 35.6% (26/73) of female patients had a malignant histology. Eighteen patients eventually needed a completion thyroidectomy.

**Conclusion:**

Compared with national data, we showed a higher risk of malignancy in Thy3 nodules in our centre. Our study should encourage other centres to audit their own data. We propose setting up a national Thy3 registry as a basis to promote research in improving preoperative diagnosis of indeterminate thyroid nodules.

## Graphical abstract

The rate of Thy3 malignancy in this study is higher compared to UK national average. This calls for regional centres in the UK to audit their local Thy3 malignancy rate and setting up of a national Thy3 registry. This could contribute to adoption of international standards and best practices.

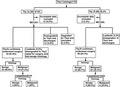

## 1 INTRODUCTION

Thyroid cancer is the most common malignant endocrine tumour but constitutes less than 1% of all malignancies treated in the United Kingdom.^1^ Although the incidence of thyroid cancer is increasing globally, the overall mortality from thyroid cancer has remained stable over many years.^2^, ^3^

Thyroid nodules are commonly seen in both, primary and secondary care. Diagnosis of benign versus malignant nodules can be challenging. Ultrasound plus or minus fine‐needle aspiration (FNA) is the gold standard used for initial diagnosis of all thyroid nodules. In the UK, thyroid nodules are assessed radiologically using the U or TI‐RADS (Thyroid Imaging Reporting and Data System) classification to determine which nodules are benign and which are suspicious requiring a FNA. Despite this, there is still a proportion of patients with indeterminate cytology (Thy3). Quoted malignancy rates in the UK for Thy3a are 5–15% and 15–30% for Thy3f.^3^ Thy3 cytology is indeterminate since follicular neoplasms require formal excision to assess capsular and/or vascular invasion within the tissue architecture to determine malignancy.^3^, ^4^

According to the statistical data resulting from the British Association of Endocrine and Thyroid Surgeons (BAETS) audit in 2012, over 50% of all thyroid surgery carried out in the UK was for benign disease.^2^ British Thyroid Association (BTA) guidelines published in 2014 for managing Thy3 nodules suggested a repeat investigation by further ultrasound assessment with or without a repeat FNA for Thy3a nodules and diagnostic hemithyroidectomy for Thy3f lesions.^3^

It can be challenging for patients to understand the ambiguity surrounding Thy3 nodules, the need for a diagnostic hemithyroidectomy, and the possibility of further treatment with surgery and radioactive iodine treatment. Therefore, preoperative counselling is key to help patients understand the risks and benefits of diagnostic thyroid surgery.

The aim of this study was to establish the risk of thyroid cancer for Thy3 nodules over five years (2015 to 2020) in our institution. The secondary aim was to compare our initial management of all Thy3 nodules against the current BTA guidelines.

## 2 MATERIALS AND METHODS

This study is a retrospective observational review of all thyroid nodules with a Thy3 cytology result investigated by ENT and endocrine departments between January 2015 and June 2020. It was conducted in a district general hospital by obtaining information from cytological reports in the pathology database. The study was registered with the clinical audit department and approved in the departmental clinical governance meeting. Since it was a retrospective audit of cytological performance with no intervention, ethical committee approval was not required.

All Thy3 nodules were discussed in a regional thyroid multidisciplinary team (MDT) meeting (Oxford University Hospitals) comprising of an oncologist, a histopathologist with specialist interest in thyroid pathology and a thyroid surgeon. Only Thy3 nodules that had a preoperative thyroid ultrasound and surgical histology were included in the study. Exclusion criteria included paucity of information on the electronic patient record (EPR) with regard to ultrasound, fine‐needle aspiration cytology or histopathology. The MDT recommendation, treatment options and risk of malignancy were discussed with each patient. This allowed the patient to make an informed choice for further management. In our practice, surgery was also offered to patients whose nodule cytology had been downgraded from Thy3 to Thy2 but measured 40 mm or greater. Additional operative criteria included compressive multinodular goitres, nodule growth on previous surveillance ultrasound imagings and/or patient preference.

During the study period, we obtained 115 patient records with Thy3 cytology between January 2015 and June 2020. We divided all Thy3 results into two main groups: Thy3a and Thy3f based on Royal College of Pathologists UK guidance (2016) document.^3^

The data set was then analysed by going through the thyroid MDT discussion on EPR to determine which results were downgraded or upgraded. All statistical analysis was performed using Microsoft Excel. Demographic characteristics were recorded for each group. The final histopathological results for each group were compared with the initial FNA results to determine the ‘rate of malignancy’. We acknowledge that our results would have an incomplete data bias due to 10 patients being excluded.

## 3 RESULTS

**FIGURE 1:**
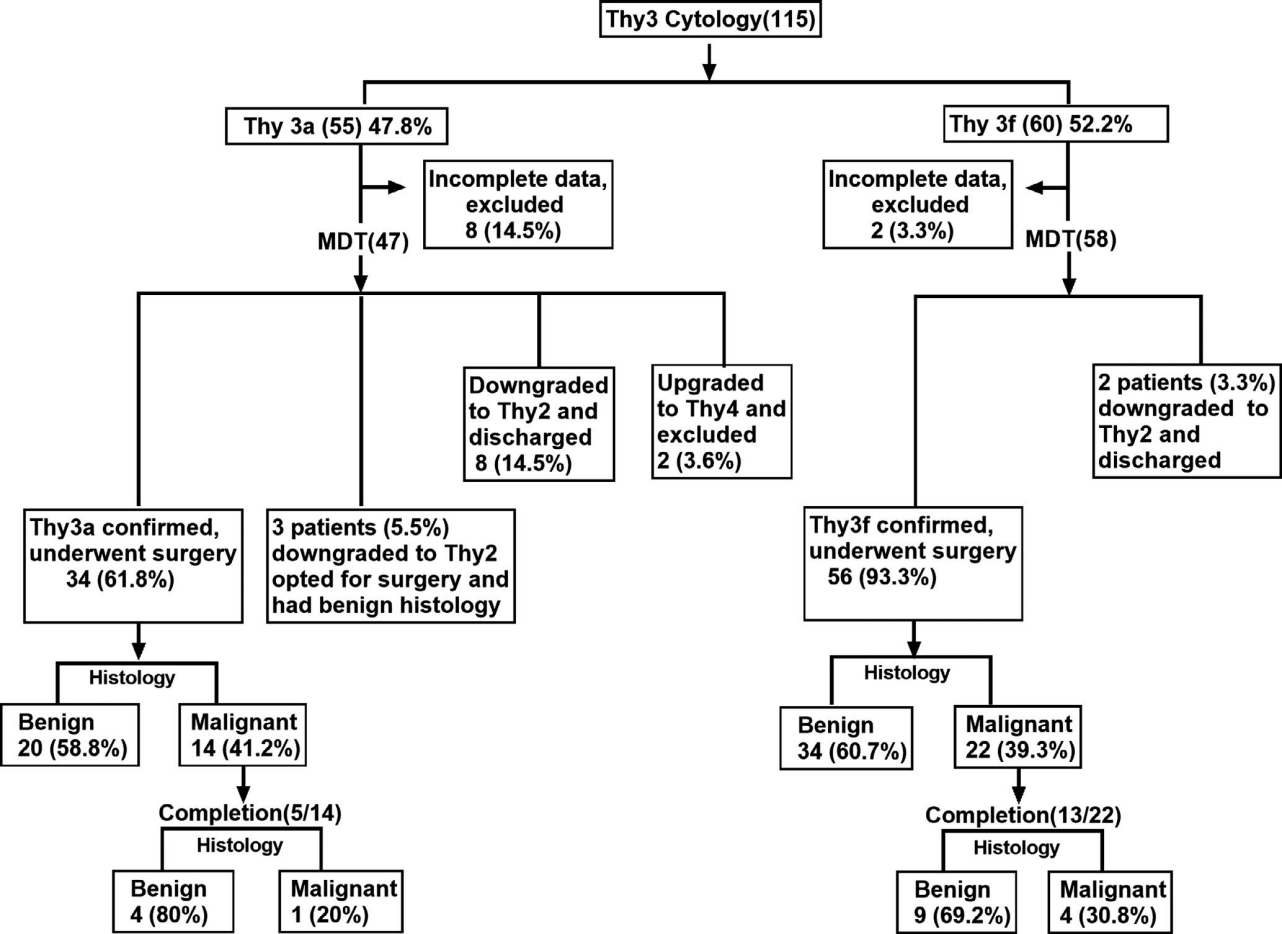
Flow chart showing the outcome of Thy3 nodules

In this study, 115 patient records were analysed. A total of 55 (47.8%) were Thy3a and 60 (52.2%) were Thy3f (Figure 1). Each cytology result was discussed in the regional thyroid MDT. Of the 55 Thy3a results, 11 (20%) were downgraded to Thy2, two (3.6%) were upgraded to Thy4 cytology, and 34 (61.8%) patients underwent surgery. Similarly, of the 60 Thy3f results, two (3.3%) were downgraded to Thy2 whereas 56 (93.3%) went ahead with surgery. A total of 10 patients had missing data (eight Thy3a and two Thy3f) and these were excluded. Overall, 13 patients were downgraded to Thy2 at MDT. Of these, 10 were discharged with no follow‐up as they previously had ultrasound surveillance of the nodule without evidence of any growth and three patients opted to proceed with surgery (patient concern). Table 1a and Table 1b shows the breakdown of ultrasound gradings for the Thy3 nodules in our study. 58.8% (10/17) of male patients and 35.6% (26/73) of female patients had a malignant histology (Table 2). The average age (in years) of patients with a malignant histology was 49 and 56.2 in the Thy3a and Thy3f groups, respectively (Table 2). Overall rate of malignancy in our study for those who underwent surgery for Thy3 lesions was 40% (36/90). The rate of malignancy in the Thy3a patients was 41.1% and 39.2% in Thy3f patients (Figure 1). All the three Thy3a patients who were downgraded at thyroid MDT but chose to undergo surgery had benign histology. The ratio of follicular thyroid carcinoma to papillary thyroid carcinoma in the Thy3f group was 11:10 compared to 8:6 in the Thy3a group. The histological outcomes for both groups have been summarized in Table 3. In our study, three patients had an incidental papillary microcarcinoma on the background of a benign goitre. 18 of the 36 patients (50%) who had a malignant histology after primary surgery underwent completion surgery (Table 4).

**TABLE 1a:** Ultrasound grading of Thy3a nodules and their outcome

US grading of Thy3a	Number of patients (n = 34)	Malignant histology	Benign histology	Malignancy risk (%)
U2	1	0	1	0
U3	17	5	12	29.4
U4	0	0	0	0
U5	0	0	0	0
TR2	0	0	0	0
TR3	3	2	1	66.6
TR4	11	6	5	54.5
TR5	2	1	1	50.0

Note

The radiology department changed from U grading to the thyroid imaging reporting and data system (TIRADS) in 2019.

**TABLE 1b:** Ultrasound grading of Thy3f nodules and their outcome

US grading of Thy3f	Number of patients (n = 56)	Malignant histology	Benign histology	Malignancy risk (%)
U2	6	2	4	33.3
U3	27	11	16	40.7
U4	5	1	4	20.0
U5	1	0	1	0
TR2	0	0	0	0
TR3	5	4	1	80.0
TR4	10	3	7	30.0
TR5	2	1	1	50.0

Note

The radiology department changed from U grading to the thyroid imaging reporting and data system (TIRADS) in 2019.

**TABLE 2:** Patient demographics in our cohort of 90 patients

	Thy3a	Thy3f
Number of patients (90)	34	56
Gender		
Male (n = 17)	3	14
Number of malignant histology in males (10/17)	1	9
Female (n = 73)	31	42
Number of malignant histology in females (26/73)	13	13
Age (years)		
Range	28–85	25–82
Average	56	55.7
Average with malignant histology outcome	49	56.2

**TABLE 3:** Histological outcomes following surgery for Thy3a and Thy3f cytology

	Thy3a	Thy3f
Benign (total)	**20**	**34**
Goitre	10	10
Goitre with thyroiditis	3	5
Follicular adenoma	5	10
Hyperplastic nodule	1	1
Hyalinizing trabecular tumour	1	0
Hurthle cell adenoma	0	4
Oncocytic adenoma	0	4
Malignant (total)	**14**	**22**
Overall PTC	**6**	**10**
Classical PTC	2	5
FVPTC	4	2
Unusual Variant of PTC	0	1
Multifocal PTC	0	1
Multifocal FVPTC	0	1
Overall FTC	**8**	**11**
FTC	1	2
Minimally Invasive FTC	5	3
Poorly differentiated FTC	2	1
FTC clear cell	0	1
Oncocytic (Hurthle) carcinoma	0	4
Hyalinizing trabecular carcinoma	0	1

**TABLE 4:** Histological outcomes in the contralateral thyroid lobe in those patients undergoing completion thyroidectomy

Malignant histology subtype from the primary surgery	PTC (7)	FTC (10)	Poorly DTC (1)
Malignant histology in contralateral thyroid lobe	1	3	0
Benign histology in contralateral thyroid lobe	6	7	1

## 4 DISCUSSION

The main objective of this study was to analyse the malignancy rates in Thy3 nodules over a five year period in our institution. The most recent guidelines on the management of thyroid cancer were issued by the British Thyroid Association in 2014.^5^ Thyroid cytology is categorized from Thy1 (non‐diagnostic) to Thy5 (diagnostic of malignancy) based on Royal College of Pathologists UK guidance document (2016).^3^ Wang et al have shown that specialist histopathology opinions regarding specific cytological subtypes such as follicular adenoma versus Hurthle cell adenoma or follicular carcinoma versus Hurthle cell carcinoma can vary from 8 to 13%.^6^ In practice, diagnosis of Thy2 and Thy5 by fine‐needle aspiration (FNA) is robust. However, interpreting Thy3 cytology has always been challenging as follicular neoplasms cannot be excluded because cytology does not show the presence of capsular and/or vascular invasion. Hence, there is wide variation in reported malignancy rates for Thy3 nodules.^6^, ^7^, ^8^

According to the Royal College of Pathologists UK guidance document (2016), Thy3a cytology confers a 5–15% malignancy rate and Thy3f diagnosis a 15–30% malignancy rate.^3^ In our study, the rate of malignancy for Thy3a was 41.1% and 39.2% for Thy3f with an overall average of 40% which is comparatively higher than the nationally quoted figure of 25.7% (fifth national BAETS UK audit report, 2017).^9^ However, according to the BTA guidelines (chapter 5)^5^ the malignancy risk quoted for Thy3 nodules can be from 9.5% to 43%. Our results for Thy3a are also comparable to the findings in a large series of 381 surgically excised indeterminate nodules (Bethesda category III) which reported a rate of malignancy of 37.8%.^10^ This reinforces that malignancy rates can be quite variable.

New thyroid cancer biomarkers have been extensively researched in recent years. Genetic, proteomic and imaging biomarkers have shown promising results with increased accuracy of preoperative diagnosis, and genetic molecular markers such as Afirma GEC^11^ and ThyroSeq v3 GC sets^12^ have made a transition into the clinic. Other recently studied markers are MPTX (ThyGeNEXT +ThyraMIR),^13^ Textural Analysis on DW‐MRI,^13^ Real Time Elastography^14^ and Conventional US + Elastography.^15^ Of these Afirma GEC showed highest sensitivity (95.5%)^11^ while Textural Analysis on DW‐MRI had highest specificity (96%).^16^ The use of molecular biomarkers has been included in the most recent iteration of the American Thyroid Association management guidelines.^10^ However, the cost‐benefit ratio has limited its universal use. The diagnostic accuracy of molecular techniques varies across different practices and requires further development.^17^

Since Thy3 nodules are indeterminate, a diagnostic hemithyroidectomy is often recommended. If the histology is benign, no further intervention is required. In our cohort, if a more accurate benign preoperative diagnosis was given using novel biological markers then up to 60% of operations may have been avoided. Thyroid surgery has associated morbidity including recurrent laryngeal nerve injury (voice change), bleeding, infection and excessive scarring. A malignant diagnosis may require further surgery in the form of completion thyroidectomy followed by adjuvant radioactive iodine ablation. Equally, if a preoperative malignant diagnosis can be made in Thy3 nodules, one definitive operation could be carried out, thereby avoiding two operations. Interestingly, thyroid tumours also consist of several pathologies which sit between a benign and malignant diagnosis, that is minimally invasive follicular thyroid carcinoma (MIFTC) and the newly classified noninvasive follicular thyroid neoplasm with papillary‐like nuclear features (NIFTP). This was observed in the retrospective study of 109 patients published by Nikiforov et al,^18^ wherein 67 patients with noninvasive encapsulated follicular variant of papillary thyroid carcinoma (NIFTP) were treated with only lobectomy and no recurrence of disease was observed at a median follow‐up of 13 years. In our study, there were eight minimally invasive FTCs but no NIFTPs. Two of the eight MIFTCs were diagnosed after a total thyroidectomy and six after a diagnostic hemithyroidectomy. Two of these went on to have a completion thyroidectomy.

Audit is a fundamental part of modern surgical practice as it helps to improve patient decision‐making and is important to quote local figures when consenting for surgery. A national registry could be set up by involving a national thyroid organization such as BAETS in the UK and trainee networks (BAETS trainee led or INTEGRATE). Funding would be needed to maintain such a registry. The registry would be open to all units across the country wishing to enter their locally audited data on annual basis. Such a registry can also be useful to audit ultrasound classification (U/TI‐RADS) and cytology (Thy) data with correlated histopathology.

### 4.1 Study limitations

Since data collection was retrospective, only those patients with histological outcome were included. Other limitations included a small cohort of 90 patients. However, most of the cytology specimens were reported or supervised by a pathologist with specialist thyroid interest, and hence, the inter‐observer variability was low.

## 5 CONCLUSION

The rate of malignancy can vary at different institutions. Hence, we recommend that all institutions audit their Thy3 malignancy rate. This rate can be used to aid the surgical consent process. The current mainstay management of Thy3 nodule starts with a discussion at the regional thyroid MDT to confirm the diagnosis. The MDT recommendation, based on the risk of malignancy, is usually a diagnostic hemithyroidectomy. In our Thy3 nodule cohort, up to 60% of operations may have been avoided if an accurate preoperative diagnosis was made using promising advances such as genetic, molecular or imaging biomarkers. Improving diagnosis of indeterminate thyroid nodules should be the focus of future research and funding. To this end, we propose setting up a national Thy3 registry which would facilitate such research. A registry should include patient demographics, tissue banks with cytology and histological specimens. Research can then be tailored to finding biomarkers which deliver the most accurate preoperative diagnosis of these nodules.

## CONFLICT OF INTEREST

The authors have no conflict of interest to declare.

## AUTHOR CONTRIBUTIONS

DN came up with the study concept, conducted the literature search, analysed data and wrote and formatted the manuscript. SK helped with data collection and contributed to drafting the manuscript. TR and RC used their clinical expertise to critique and edit the manuscript. SN oversaw the data analysis and helped prepare, edit and revise the manuscript until the final version. All authors have read and approved the final manuscript.

## Data Availability

Data available on request from the authors.
